# Correction: Quantum Entanglement and Spin Control in Silicon Nanocrystal

**DOI:** 10.1371/annotation/e78f3177-4e7e-4c2b-8c42-797234af665c

**Published:** 2013-10-24

**Authors:** Vesna Berec

The number of the grant from the Ministry of Science, Republic of Serbia was incorrectly given. The Funding Statement should read: "This work is supported by the Ministry of Science, Republic of Serbia. The funders had no role in study design, data collection and analysis, decision to publish, or preparation of the manuscript." 

